# Novel VEGF Decoy Receptor Fusion Protein Conbercept Targeting Multiple VEGF Isoforms Provide Remarkable Anti-Angiogenesis Effect *In Vivo*

**DOI:** 10.1371/journal.pone.0070544

**Published:** 2013-08-12

**Authors:** Qin Wang, Tao Li, Zhigang Wu, Quan Wu, Xiao Ke, Delun Luo, Hui Wang

**Affiliations:** 1 State Key Laboratory of Pathogens and Biosecurity, Beijing Institute of Microbiology and Epidemiology, Beijing, P. R. China; 2 Chengdu Kanghong Biotechnology Co. Ltd., Chengdu, P. R. China; National Center for Cell Science, India

## Abstract

VEGF family factors are known to be the principal stimulators of abnormal angiogenesis, which play a fundamental role in tumor and various ocular diseases. Inhibition of VEGF is widely applied in antiangiogenic therapy. Conbercept is a novel decoy receptor protein constructed by fusing VEGF receptor 1 and VEGF receptor 2 extracellular domains with the Fc region of human immunoglobulin. In this study, we systematically evaluated the binding affinity of conbercept with VEGF isoforms and PlGF by using anti-VEGF antibody (Avastin) as reference. BIACORE and ELISA assay results indicated that conbercept could bind different VEGF-A isoforms with higher affinity than reference. Furthermore, conbercept could also bind VEGF-B and PlGF, whereas Avastin showed no binding. Oxygen-induced retinopathy model showed that conbercept could inhibit the formation of neovasularizations. In tumor-bearing nude mice, conbercept could also suppress tumor growth very effectively in vivo. Overall, our study have demonstrated that conbercept could bind with high affinity to multiple VEGF isoforms and consequently provide remarkable anti-angiogenic effect, suggesting the possibility to treat angiogenesis-related diseases such as cancer and wet AMD etc.

## Introduction

The pathological angiogenesis is a critical hallmark of human diseases such as the cancer [1] and the wet form of age-related macular degeneration (AMD) [2] – the leading cause of blindness in the elderly population. Compelling evidences have demonstrated that vascular endothelial growth factor (VEGF) plays a pivotal role in the abnormal angiogenesis process [3]. Therefore VEGF has become a key target in antiangiogenic therapy [4]. There are five members in VEGF family: VEGF-A, -B, -C, -D and placental growth factor (PlGF) [5]. Among them, VEGF-A is the first discovered and the most well-studied member [6]. Alternative exon splicing and proteolytic cleavage generated several distinct VEGF-A isoforms, respectively named VEGF121, VEGF165, and VEGF162. VEGF-A isoforms are all active as dimers, differing principally in their size and ability to bind heparin or neuropilins. Isoforms of VEGF-B and PlGF, which differ in their capacity to bind heparin, are also produced by alternative splicing.

Several therapeutic drugs against VEGF isoforms and PlGF have been developed by different companies. Pegaptanib (Macugen™, Eye- tech, Inc.) was the first anti-VEGF drug approved for the treatment of wet AMD [7], [8]. Bevacizumab (Avastin®, Genentech, Inc.) is a recombinant humanized monoclonal antibody [9], which has been approved by the US Food and Drug Administration (FDA) for the treatment of metastatic colorectal cancer, non-small-cell lung cancer, glioblastoma [10]. And ranibizumab (Lucentis®, Genentech, Inc.), a Fab fragment similar to Bevacizumab, has been also approved for the treatment of wet AMD [11].VEGF Trap (aflibercept, Regeneron Pharmaceuticals, Inc.) is a novel soluble decoy receptor generated with Trap technology. It employs the fusion of components from multiple endogenous receptors [12]. Although pharmacological inhibition of VEGF-A has proven to be effective in inhibiting angiogenesis and vascular leak associated with cancer and various eye diseases [13], presently, little information is reported on the binding kinetics and affinity of various VEGF inhibitors.

**Figure 1:**
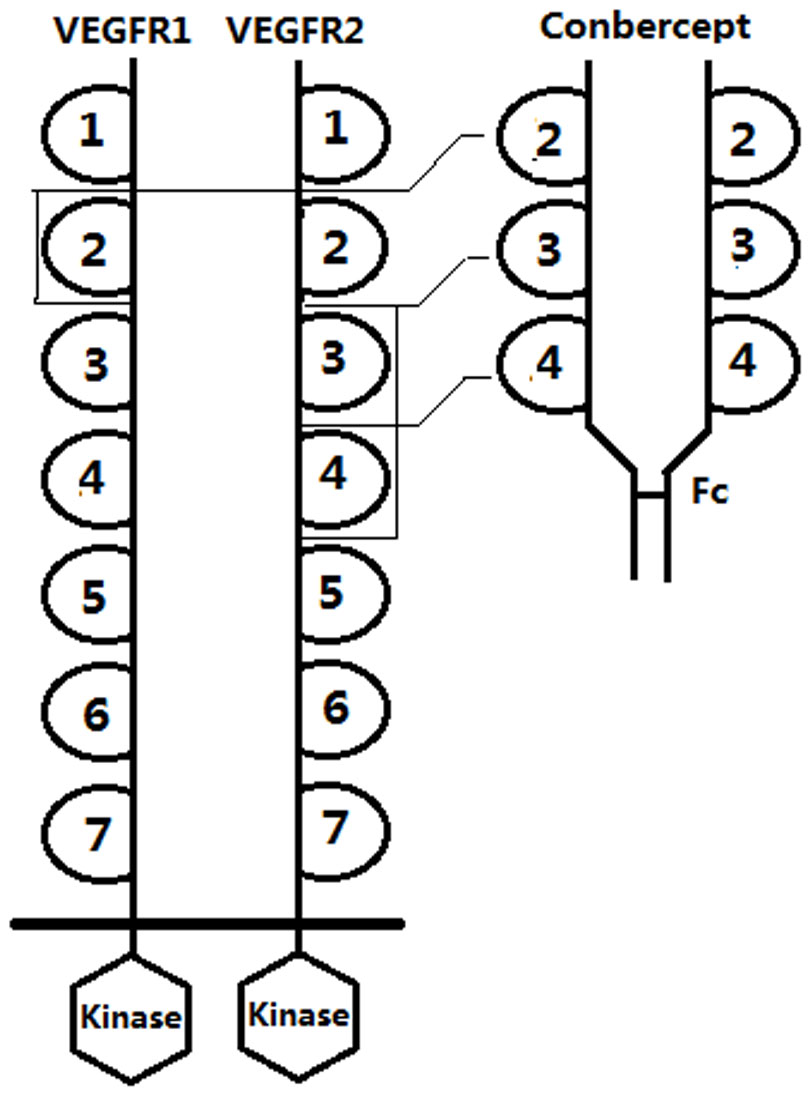
Schematic structure of conbercept. VEGF receptor-1 and receptor-2 are related receptors that have seven extracellular Ig domain plus an intracellular tyrosine kinase domain. Conbercept contains the Ig domain 2 of VEGF receptor-1 fused to the Ig domain 3 and 4 of VEGF receptor-2 fused to the IgG1 Fc.

Conbercept is a recombinant fusion protein composed of the second Ig domain of VEGFR1 and the third and fourth Ig domain of VEGFR2 to the constant region (Fc) of human IgG1 (Figure 1).The structural characterization of conbercept was reported not long ago [14]. It is designed as a receptor decoy with high affinity for all VEGF isoforms and PlGF. The inhibitory effects of conbercept to VEGF have been evaluated in vitro and vivo, indicating that conbercept exerts potent anti-angiogenic and anti-tumor effects [15]. The surface plasmon resonance (SPR) biosensor system is widely used to determine binding affinity between proteins due to the advantages of direct and label-free detection with dispersionless microfluidics [16]. In a previous work, based on BIACORE 3000 instrument (Uppsala, Sweden; GE), we have assessed the pre-clinical immunogenicity on the rhesus monkeys administrated with intravenous conbercept [17]. Subsequently, in the phase I clinical trial, we continued to detect the serum immunogenicity on the cancer and AMD patients cured with conbercept (data to be published). In this study, we not only evaluated the kinetics and affinity characteristics of conbercept and Avastin with VEGF isoforms using Biacore and ELISA, but proved the anti-angiogenic effects of conbercept in choridal neovasularization (CNV) growth and permeability experiments by animal models. These results strongly support the pre-clinical and sequential phase clinical trials of conbercept.

## Materials and Methods

### 1. Reagents

The conbercept protein used in this study was produced from Chinese hamster ovary cells and supplied by Chengdu Kanghong Biotechnology Co. Ltd. (Chengdu, China, 610036). VEGF-A165, VEGF-A121, VEGF-B167 and PlGF were purchased from R&D Company. CM5 microchips and Amine Coupling Kit were obtained from GE. ELISA reagents were purchased from BETHYL and R&D. 5-FU was purchased from Tianjin Jinyao Amino Acid Co. Ltd. M200 medium and low serum growth supplement (LSGS) used for the growth of human umbilical vein endothelial cells (HUVEC) were ordered from Cascade (Carlsbad, CA, USA).

### 2. The binding efficiency analysis by ELISA assay

ELISA: Antigens (VEGF-A 165 or VEGF-B 167 or PlGF) were diluted to a final concentration of 4 nM in PBS. The wells of a PVC microtiter plate were coated with the antigen by pipeting 100 µl of the antigen dilution in the top wells of the plate and incubated at 4°C overnight. The coating solution was removed and the plate was washed three times by filling the wells with 200 µl PBS. The remaining protein-binding sites were blocked by adding 200 µl blocking buffer containing 1%BSA in PBS and then the plate was incubated at 37°C for 2 hours. The plate was washed three times by filling the wells with 200 µl PBS. 100 µl of diluted primary antibody (conbercept or Avastin) was added into each well, the plate was covered with an adhesive plastic and incubated for 2 h at room temperature. The plate was washed four times with PBS. 100 µl of conjugated goat anti-human secondary antibody labeled with horse radish peroxidase (HRP) was added, diluted at 1∶50000 in blocking buffer before use. The plate was covered with an adhesive plastic and incubated for 1 h at 37°C. The plate was washed four times with PBS. 100 µl of the substrate solution was dispensed per well. After 10 min incubation at 37°C, 100 µl of stop solution was added into the wells. The absorbance of each well was read with a plate reader at 450 nM as the detection wave length.

### 3. Kinetics and Affinity detection by BIACORE

The kinetics and affinity of conbercept with VEGF isoforms were analyzed by surface plasmon resonance technology using a BIACORE 3000 instrument (Uppsala, Sweden, GE). VEGF-A isoforms and PlGF (in 10 mM sodium acetate) were set as ligands to be immobilized onto the CM5 biosensor microchips by the amine coupling method using N-ethyl-N-dimethylaminopropyl carbodimide and N-hydroxy-succinimide. About 5,000±500 response units (RUs) per flow cell were immobilized to the channel FC2-4, and the FC1 channel was set as the blank control. Microchip sensor surfaces were conditioned with three cycles of five injections of 5 µl of 15 mM HCl, followed by three injections of 10 µl of 1.0 M NaCl, and finally by three cycles of five injections of 10 µl of HBS buffer (10 mM HEPES, 0.15 M sodium chloride, 3.4 mM EDTA, and 0.005% surfactant P20) prior to the kinetics and affinity analysis. The affinity detective process was performed by the written wizard program of the BIAcontrol software. The kinetic curve and affinity data were acquired by injecting conbercept and Avastin onto the CM5 chips immobilized with VEGF isoforms. Microchip sensor surfaces were regenerated with 10 µl of 20 mM NaOH prior to every new injection cycle enabling microchip surfaces to be stable for 30–40 cycles. The alterations in the refractory index were recorded as relative response unit (RU) as described by the manufacturer (Uppsala, Sweden, GE). The dissociation rate constant (kd), the association rate constant (ka), the dissociation equilibrium constant (KD), and the association equilibrium constant (KA) were automatically calculated with the BIAevaluation Software3.0 (Uppsala, Sweden, GE).

### 4. Endothelial Cell Proliferation Assay

The proliferation of HUVEC is in response to recombinant VEGF165, which can bind conbercept and Avastin selectively. Thus the potency of conbercept and Avastin can be measured based on the ability to inhibit the proliferation of HUVEC.

To measure proliferation, HUVEC cells were thawed and resuspended at 2×104 cells/ml in ECM with growth factor and FBS supplementation. About 100 µl of the cells suspension was seeded into each well of a 96-well tissue culture plate and the growth medium in each well was aspired after 24 hours. For conbercept and Avastin inhibition, serial dilutions of conbercept and Avastin were mixed with 40 ng/ml of VEGF165 at 37°C for 3 hours. 100 µl of the mixture was then added to HUVEC cells per well after aspirated supernatant. The plate was continuously incubated at 37°C with 5% CO2 for 4 days. At the end of incubation, 20 µl of CCK-8 was added to each well and the plate was incubated for an additional 4 hours under the same condition. Then the plate was read at 490 nm on a plate reader.

### 5. Animals and ethics statement

All necessary permits were obtained for the animal experiments. This study was approved by Institutional Ethics Review Committee of Beijing Institute of Microbiology and Epidemiology, China. All procedures on the animals were carried out in strict accordance with the regulations of the Beijing Institute of Microbiology and Epidemiology Animal Care and Use Committee (2010215). Animals were maintained under specific pathogen-free conditions (25∼28°C, relative humidity 55±15%, 12-hr light/dark cycles). All animals were handled under the care and supervision of a veterinarian. The mice were sacrificed by cervical dislocation at the end of the experiment.

### 6. Oxygen-Induced Retinopathy Suppression Experiments

Ten KunMing timed-pregnant mice were obtained from Sichuan University laboratory animal center and were housed in the animal care facilities of Sichuan University. In the newborn mouse model of oxygen-induced retinopathy (OIR), 7-day-old mice were placed with their maternal mice in a (75±3)% oxygen atmosphere for 5 days (D7–D11), and then returned to normoxia for further treatment.

The neonatal mice were divided into 3 groups. Group 1 was set as normal control. Group 2 and 3 were OIR models, treated with normal saline and conbercept (25 mg/kg) (40 µL) intraperitoneally every other day. All animals were euthanatized on D21. Vascular perfusion of fluorescein (FITC-dextran) and retinal stretched preparation were used to observe the retinal vessels. The sections of eyeballs with H&E staining were used for counting the endothelial cells of new vessels beyond the inner limiting membrane under the microscopy (6 µm-thin sections were cut sagittally parallel to the optic nerve, and 38 sections were extracted discontinuously from an eyeball) [18].

### 7. Tumor Growth Experiments

Fifty-six female BALB/c nude mice (5∼8 w, 16∼18 g) were obtained from Shanghai SLRC laboratory animal Co. Ltd. Human colon cancer cells (LOVO) were injected subcutaneously in the axilla (1×10^7^ cells/0.2 ml each mouse) at the start of the study. The animals were divided into 7 groups, each group had 8 mice. Group 1 received normal saline (NS) as a control. Group 2–4 received conbercept at 2, 6 and 18 mg/kg, respectively. Group 5 received Avastin at 6 mg/kg. Group 6 received 5-FU at 50 mg/kg. Group 7 received 5-FU (50 mg/kg) and conbercept (6 mg/kg). Dosing began 24 hours after tumor implantation. Each animal received injections of test material twice a week for a total of 10 doses. The tumors (big enough to be measured by vernier caliper) were measured weekly till week 6. The evaluation criteria were tumor weight,tumor volume and anti-tumor rate. At the end of study, the tumors were weighted after sacrifice of the animals. Tumor volume (TV) = a×b2/2 (a, b means the length and width of the tumor). Anti-tumor rate(%) = (The mean tumor weights of negative control group – The mean tumor weights of treatment group)/The mean tumor weights of negative control group×100%.

### 8. Data availability statement

The methods used in this paper were validated for selectivity, linearity, accuracy, precision, recovery and stability according to the FDA guidelines for the validation of bioanalytical methods (USFDA, http://www.fda.gov/downloads/Drugs/Guidance Compliance Regulatory Information/Guidances/ucm070107.pdf, 2001).

### 9. Statistics

Statistical analysis was carried out using SPSS version 11.0 (SPSS Inc., Chicago, Illinois, USA). All results are expressed as the mean ± standard deviation. The comparison of baseline OD value was performed by Student's t test. A probability of P<0.05 was considered to be statistically significant.

## Results

### 1. The binding efficiency comparison of conbercept and Avastin with VEGF isoforms and PlGF by ELISA

**Figure 2:**
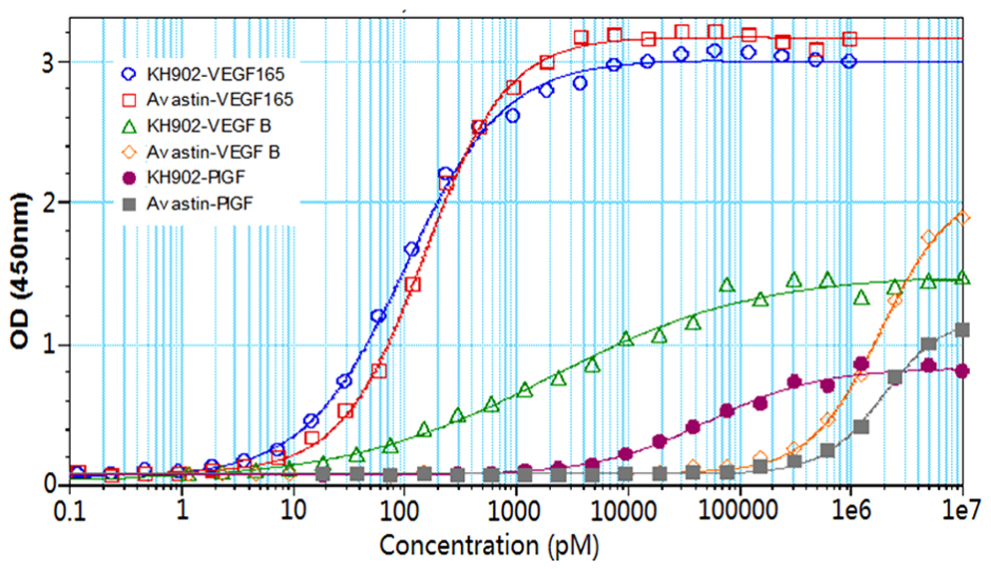
The binding efficiency comparison of conbercept and Avastin with VEGF isoforms and PlGF by ELISA. Conbercept present in figure by using an alias as KH902.

The binding specificity and efficiency of conbercept and Avastin with VEGF isoforms and PlGF were first examined by ELISA (Figure 2). In this experiment, both conbercept (EC_50_, concentration for 50% of maximal effect, EC50 = 0.1 nM) and Avastin (EC50 = 0.15 nM) showed high binding affinity with VEGF-A 165. Conbercept binds VEGF-B and PlGF with relative affinity of 2 nM and 52 nM respectively, whereas Avastin shows no detectable binding to VEGF-B and PlGF. Avastin, as a human antibody against VEGF-A, is not expected to bind other VEGF isoforms. However, conbercept composed of portions of VEGF receptors, is expected to bind VEGF-B and PlGF as well.

### 2. The affinity comparison of conbercept and Avastin with VEGF-A isoforms by Biacore

**Figure 3:**
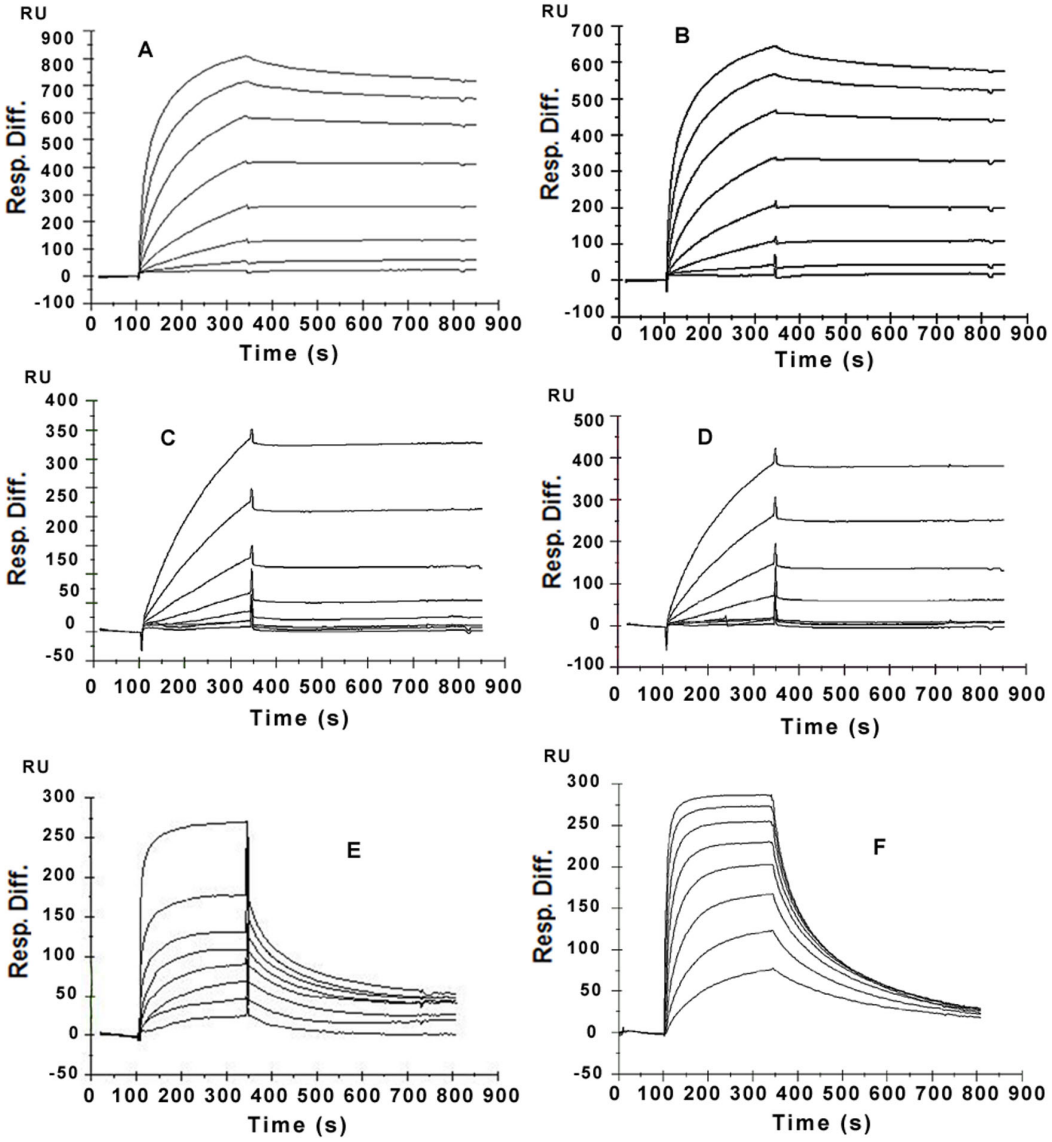
The Dynamic Curves of VEGF inhibitors with VEGF isoforms and PlGF detected by BIACORE. VEGF isoforms and PlGF were set as ligands to be immobilized onto the CM5 chips. Different VEGF inhibitors were set as analytes and two-fold diluted into six or eight concentrations. (A) conbercept with VEGF-A165 (B) conbercept with VEGF-A121. (C) Avastin with VEGF-A165. (D) Avastin with VEGF-A121. (E) conbercept with VEGF-B167. (F) conbercept with PlGF.

VEGF-A isoforms were set as ligands to be immobilized to the channel FC2-4. The FC1 channel was set as the blank control. The final curves and data results were automatically presented as FC2-1, FC3-1, and FC4-1 by the BIAevaluation Software3.0. Conbercept and Avastin were set as analytes to be injected onto the surface of CM5 biosensor microchips immobilized by VEGF-A isoforms. The analytes were all two-fold diluted into eight concentrations with HBS buffer ranging from 1.25 nM to 160 nM. The dynamic reactive process between analytes and ligands was automatically recorded by the BIAcontrol software. The curve and data results were analyzed by the BIAevaluation 3.0 software according to the 1∶1 binding with baseline drift model. The dynamic association and dissociation curves were displayed from Figure 3A to Figure 3D. The kinetics and affinity constants between analytes and ligands were listed in Table 1. The data indicated that conbercept had high affinity to VEGF-A165 (5.4 nM) and VEGF-A121 (5.26 nM). As reference, Avastin showed binding affinity to VEGF-A165 (24.3 nM) and VEGF-A121 (20.3 nM).

**Table 1:** The detected kinetics constants comparison of different VEGF inhibitors with VEGF isoforms and PlGF.

Analytes	Kinetics Constant	Ligands			
		VEGF-A165	VEGF-A121	VEGF-B167	PlGF
**Conbercept**	ka (1/Ms)	1.94e5	1.99e5	6.83e4	1.14e5
	kd (1/s)	1.05e-3	1.05e-3	5.58e-3	5.81e-3
	KD (M)	5.40e-9	5.26e-9	8.18e-8	5.12e-8
**Avastin**	ka (1/Ms)	2.67e4	3.40e4	NA	NA
	kd (1/s)	6.48e-4	6.88e-4	NA	NA
	KD (M)	2.43e-8	2.03e-8	NA	NA

NA: No binding data was acquired by Biacore.

### 3. The multi-targets binding ability of conbercept with VEGF-B167 and PlGF

VEGF-B167 and PlGF were set as ligands to be immobilized to the channel FC2-4. The FC1 channel was set as the blank control. The final curves and data results were automatically presented as FC2-1, FC3-1 and FC4-1 by the BIAevaluation Software3.0. Conbercept was set as analyte to be injected onto the surface of CM5 biosensor microchips immobilized by VEGF-B167 and PlGF. Conbercept was two-fold diluted into eight concentrations with HBS buffer ranging from 31.25 nM to 4000 nM. The dynamic curves of conbercept with VEGF-B167 and PlGF were recorded in Figure 3E, 3F. Biacore data showed that the KDs of conbercept with VEGF-B167 and PlGF were 81.8 nM and 51.2 nM, respectively, about 10 times lower than it with VEGF-A isoforms.

### 4. Comparison of bioactivity between Avastin and conbercept

**Figure 4:**
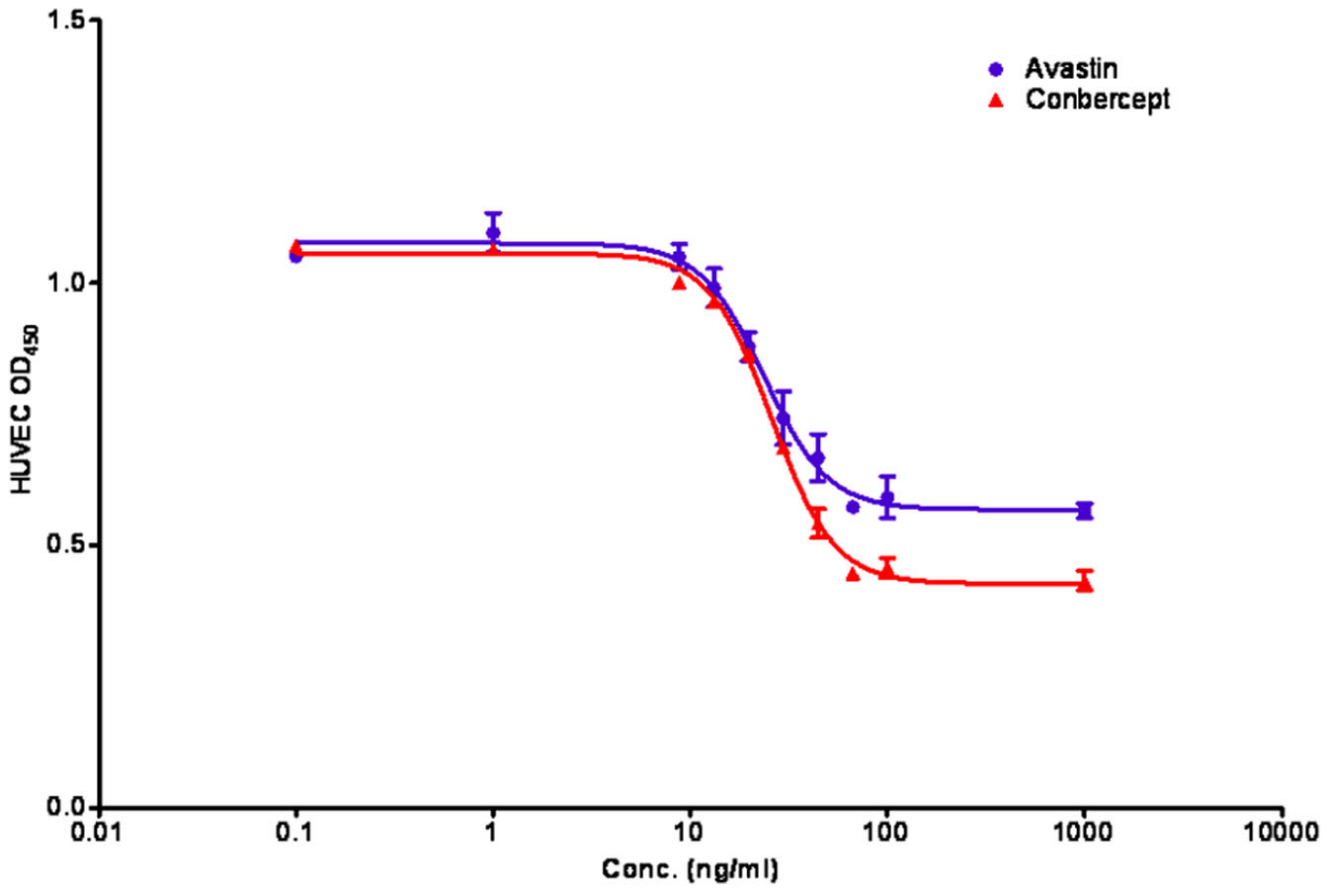
Endothelial Cell Proliferation Assay of conbercept and Avastin.

The growth of endothelial cells is induced by a lot of factors including VEGF. To determine whether conbercept binding with VEGF could effectively block the ability of VEGF-induced endothelial cells proliferation. The inhibitory effect of conbercept was evaluated by endothelial cell proliferation assay. Both Avastin and conbercept could completely block VEGF-induced HUVEC growth at the concentration of 100 ng/ml. Conbercept had a lower plateau than Avastin, as shown in Figure 4, probably due to higher inhibition potency.

### 5. Inhibition of oxygen-induced CNV growth and permeability

**Figure 5:**
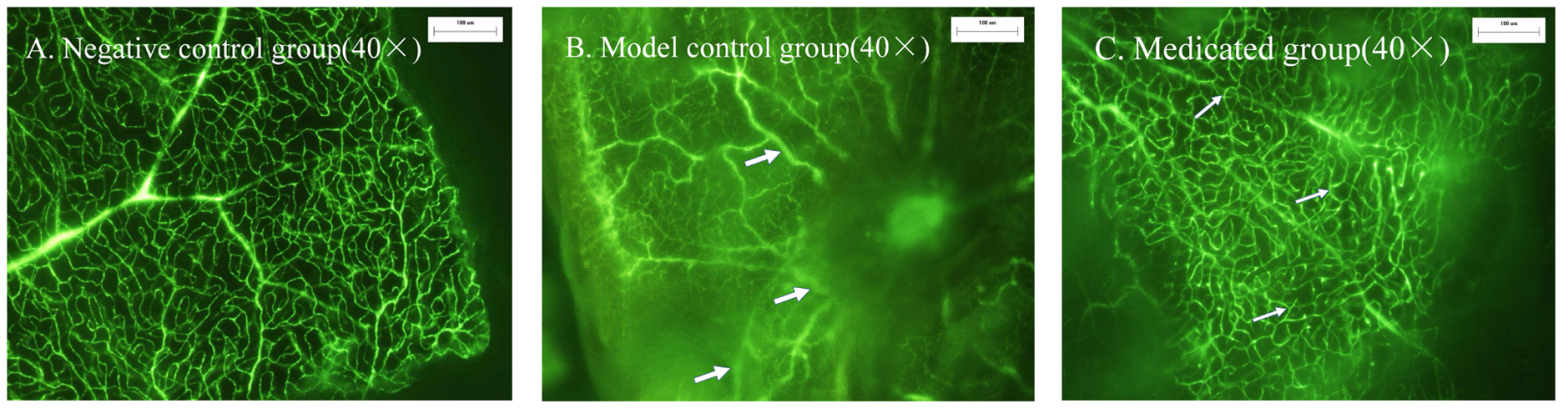
FFA results of Hypoxic ischemic retinopathy Suppression Experiments. Heavy leakages were observed indicating OIR models successfully established (the arrow pointing in the Figure 5B). Less leakage and fewer neovasularizations were observed in OIR models treated with conbercept (the arrow pointing in the Figure 5C). (A) Negative control group. (B) Model control group. (C) Medicated group.

There was no permeability and CNV detectable in the normal group. Heavy leakages were observed in OIR models treated with normal saline (NS) indicating the generation of new vessels. Whereas, less leakage and fewer neovasularizations were observed in OIR models treated with conbercept (Figure 5). The results of H&E staining also showed that the number of vascular endothelial cells beyond the inner limiting membrane was less in conbercept treated group than NS treated group(p<0.01) (Table 2).

**Table 2:** The numbers of vascular endothelial cells beyond the inner limiting membrane (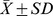).

Group	Number of eyeball	Number of sections	Number of vascular endothelial cells beyond the inner limiting membrane	Number of vascular endothelial cells beyond the inner limiting membrane in a section
Group 1	10	380	15	0.04±0.20
Group 2	10	380	14060	37±12.20*
Group 3	10	380	1900	5±2.80* Δ

Group 1: Normal control; Group 2: OIR models treated with NS; Group 3: OIR models treated with Conbercept.

* Statistical differences were significant compared to group 1 (p<0.01);

Δ Statistical differences were significant compared to group 2 (p<0.01).

### 6. Anti-tumor effects of conbercept in vivo

**Figure 6:**
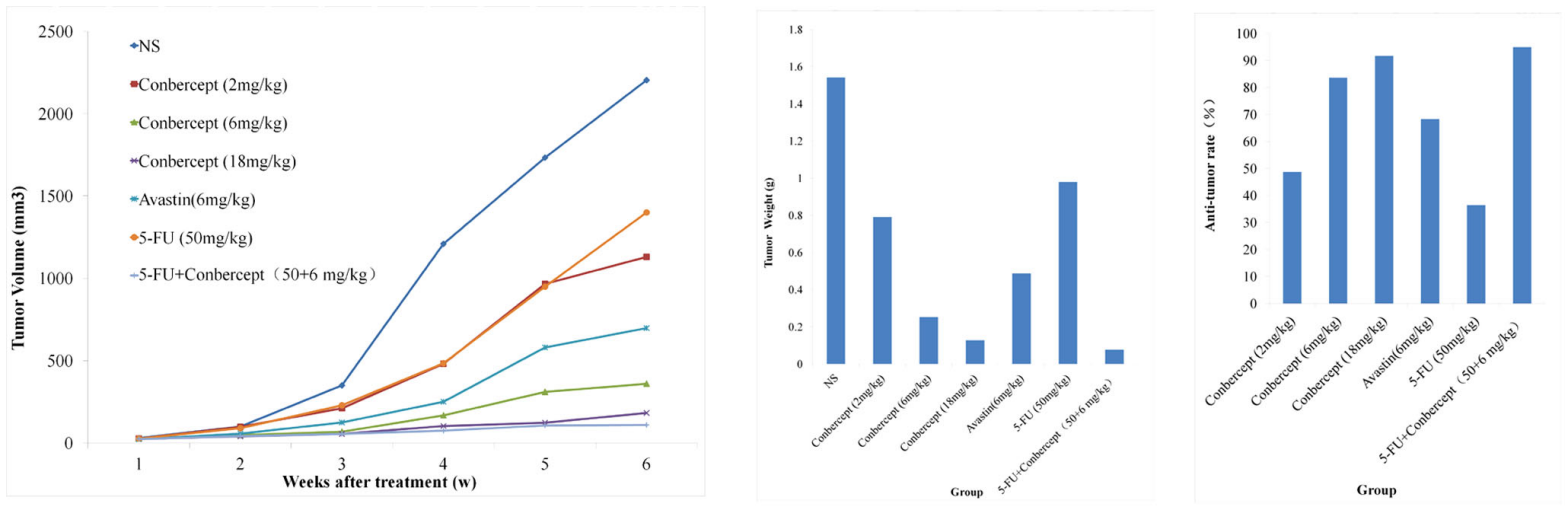
The growth dynamics of LOVO in nude mice. The tumor volume was measured weekly through 6 weeks. The figure showed the mean TV per group at different time points after tumor implantation. Group1: normal saline (NS). Group 2–4: conbercept at 2, 6 and 18 mg/kg, respectively. Group5: 6 mg/kg Avastin. Group 6: 5-FU at 50 mg/kg. Group 7: 5-FU (50 mg/kg) and conbercept (6 mg/kg). (A) Tumor volume in each group. (B) The mean tumor weights at the end of experiment. (C) Anti-tumor rates (%).

There was a sigmoidal relationship between the mean tumor volume per group and time after tumor implantation (Figure 6A). Tumor volumes in all treatment groups were significantly smaller than in the control group (p<0.05).

Compared with the NS group, the tumor weights in the low, middle and high Conbercept dosage groups became obviously lower, which were 0.791 g, 0.253 g and 0.128 g respectively (Figure 6B). The positive control Avastin group was 0.488 g. The tumor weight was 0.98 g in the group given 5-FU, in contrast, the group given 5-FU plus conbercept was only 0.077 g.

Anti-tumor rates were calculated according to the formula mentioned in the method section. The anti-tumor rates were 48.70%, 83.63% and 91.73% respectively in the low, middle and high conbercept dosage groups, indicating dose-dependent anti-tumor effect. The anti-tumor rate was 36.46% in the group given 5-FU, as compared with 94.98% in the group given 5-FU plus conbercept (Figure 6C). As positive control, Avastin showed 67.85% anti-tumor rate.

## Discussion

Many studies have demonstrated that VEGF family factors, especially VEGF-A isoforms, are the major promoters of pathological angiogenesis process characterized by an increase in the number of proliferating endothelial and stromal cells, and altered morphology of the vasculature [19], [20]. Abnormal angiogenesis does not stimulate tumor growth, but could cause vascular leak in the ocular vascular diseases such as the wet AMD and ischemic retinopathies [21]. Therefore, it has been important strategy to find blockers of VEGF isoforms for cancers and eye diseases therapy.

For a therapeutic drug, the binding affinity, half-life, bioavailability, molecular size, stability, and effectiveness should be evaluated [22]. Above all, the binding affinity is the key of the drug's efficacy. The equilibrium dissociation constant (KD) of Avastin for VEGF-A was 1.8 nM [23] or 20 nM [24] determined by SPR technology (Biacore). Recently, Wiegand *et al.* reported that the KD of Lucentis for VEGF- A165 was 46 pM. In contrast, KD of VEGF Trap was less than 1 pM for VEGF-A121 and VEGF-A165 [25].

Besides VEGF, some other factors are also involved in abnormal angiogenesis, such as PlGF and angiopoietins [26]. Researchers have demonstrated that the drugs with high binding affinity with multiple antigens exhibit the most effective blockade of tumor angiogenesis and vascular leakage, and thereby prevent tumor growth and metastasis. In this study, we employed Biacore and ELISA assays to study the kinetic and affinity of conbercept with VEGF isoforms and PlGF. Both technologies showed that conbercept could bind VEGF-A, VEGF-B and PlGF whereas Avastin could only bind VEGF-A. The Biacore result of Avastin with VEGF-A was in close agreement with earlier reports [24], but different from the other research [25]. It is probably due to different experimental setups from study to study. Only in the same experiment, the relative values of the binding affinity are considered comparable. In previous study, conbercept was examined for the inhibitory effect in the choroidal neovascularization (CNV) monkey model. It has been proved that Conbercept can effectively inhibit leakage and growth of the CNV in rhesus monkeys without evidence of toxicity [27]. In this study, we proved that conbercept present high inhibitory activity on the proliferation of human umbilical vein endothelial cells (HUVECs) induced by VEGF. Oxygen-induced retinopathy model also demonstrate the anti-angiogenic activity of conbercept. The results indicated that conbercept could suppress the formation of CNV, which was confirmed by the histological assessment, and thus could reduce vascular leakage subsequently. We concluded that VEGF and PlGF may play an important role in mediating the formation of new vessels, so conbercept could be effective in the animal models of retinopathy.

Furthermore, tumor-bearing nude mice were used to evaluate the biological activity of conbercept in vivo. As one of the most successful antiangiogenic drugs, Avastin has been proven to be effective in many preclinical and clinical studies [28], [29]. And in this study, Avastin was taken as reference to evaluate effectiveness of biological activity of conbercept in vivo. The results showed that tumor suppression was dose-dependent and could be achieved at each dose level. The middle-dose (6 mg/kg) conbercept plus 5-FU showed the best efficacy, in agreement with the clinical data: combination of antiangiogenesis with chemotherapy was more effective in cancer treatment. The possible reason is that the antiangiogenic drugs induce functional normalization of tumor vasculature resulting in tumor cell exposuring to co-administered cytotoxic drugs [30]. In this study, 6 mg/kg of conbercept showed better anti-tumor activity than the same dosage group of Avastin (p<0.05). One possible reason is that conbercept can effectively block VEGF with higher affinity in human colon cancer cells or in mice. Another possible reason is that conbercept can also bind PlGF and VEGF-B other than VEGF-A. Although the main function of conbercept was to block VEGF-A, the slight suppression of VEGF-B and PlGF might assist the action of anti-VEGF-A according to previous articles [31]. Therefore, it might be more active for the treatment of cancer than anti-VEGF-A antibody.

In summary, as a novel chimeric decoy receptor, conbercept can bind to multiple targets (VEGF-A, VEGF-B and PlGF) for antiangiogenic therapy and consequently suppress tumor growth very effectively in vivo. These attributes give strong support for the preclinical and further clinical trials of conbercept, and can be beneficial in treating various ocular disorders and cancers.
